# Study on the clinical efficacy of soothing moisturizing repairing cream combined with desonide in the treatment of atopic dermatitis in children

**DOI:** 10.1097/MD.0000000000041277

**Published:** 2025-01-17

**Authors:** Yuping Zhang, Yueqing Chen, Chen Li

**Affiliations:** a Department of Dermatology, Zhongshan City People’s Hospital, Zhongshan, Guangdong, China; b Guangdong Medical University, Zhanjiang, Guangdong, China.

**Keywords:** atopic dermatitis, children, clinical efficacy, desonide, soothing moisturizing repairing cream

## Abstract

This study evaluates the efficacy and safety of soothing moisturizing repairing cream combined with desonide in the treatment of atopic dermatitis (AD) in children in the real world. A total of 37 children with AD were randomly divided into experimental (17) and control (20) groups. For the experimental group, treated with moisturizing cream combined with desonide cream, the maintenance period was with moisturizer alone. For the control group, treated solely with desonide, the maintenance period was with nothing. Clinical assessments were made before and 1, 2, and 4 weeks after treatment. Efficacy indicators are compared between the 2 groups, and the relieving effect of moisturizer on the maintenance period of AD in children is observed. Adverse reactions are also recorded. After 4 weeks of treatment, the cure rate and effectiveness rate in the experimental group were 52.9% and 100%, respectively, compared to 0% and 60% in the control group; the estimated median maintenance time was 48 days for the control group and 73 days for the experimental group. The incidence of adverse reactions shows no statistical significance. The combination of moisturizing cream and desonide in the treatment of AD in children is highly effective and safe. It is worth promoting for clinical application due to its significant therapeutic effects and high safety profile.

## 1. Introduction

Atopic dermatitis (AD), also known as atopic eczema, is a chronic, recurrent inflammatory skin disease, often beginning in infancy, with a course that can extend into adulthood for some patients.^[1]^ Approximately 15% to 20% of children and 1% to 3% of adults worldwide suffer from AD. In China, the prevalence of AD is increasing, predominantly affecting children. Without treatment, AD can adversely impact a child’s growth and psychosocial health, increasing the burden on families and caregivers. A 2014 survey in 12 Chinese cities revealed a prevalence of 12.94% among children aged 1 to 7 years.^[2]^ The clinical manifestations of AD vary across different age stages, typically classified into infant, childhood, and adolescent to adult phases. In infants (birth to 2 years), commonly referred to as “milk scurf,” the initial lesions are itchy erythema on the cheeks, progressing to small papules and vesicopapules, which can become eroded, exudative, and crusted due to scratching and rubbing; these usually improve or resolve by age 2, although some cases may progress to childhood AD. According to statistics, the prevalence rate of infants and young children before the age of 1 is as high as 30.48%^.[3]^ In childhood (2–12 years), AD often worsens 1 to 2 years after infantile AD subsides, with lesions commonly affecting the flexural areas of the limbs, and common sites of involvement in children under 10 including the cheeks, antecubital and popliteal fossae, anterior ankle, and neck.

The pathogenesis of AD involves both genetic and environmental factors, with impaired skin barrier function and skin dryness being key factors in its development.^[4]^ Repairing the skin barrier can aid in the treatment of AD.^[5]^ The choice of treatment for AD largely depends on the patient’s age, the severity of the condition, the medical facilities available, and the patient’s economic status. Clinically, treatment of childhood AD primarily involves the use of mild to moderate potency corticosteroids.^[6]^ In recent years, it has been commonly accepted that for infants and young children with AD, using moisturizers as a basic treatment strategy, in conjunction with corticosteroids, yields better clinical outcomes than corticosteroids alone. Several studies have also highlighted the utility of new oral agents, such as Janus kinase inhibitors, as valuable options for treating pediatric AD. Hagino et al^[7,8]^ showed that the Janus kinase 1 inhibitor upadacitinib can effectively reduce ADCT (Atopic Dermatitis Control Tool) and EASI (Eczema Area and Severity Index) scores by week 4, with no serious adverse effects reported up to week 48 of treatment.

This study is designed as a prospective real-world study, employing a double-blind randomized controlled trial to observe the efficacy of desonide cream alone and the combination of soothing moisturizing repairing cream with desonide cream in treating children with AD, aiming to provide a more effective and safer treatment option for AD. The report is as follows.

## 2. Materials and methods

### 2.1. Study design

**Figure 1.**
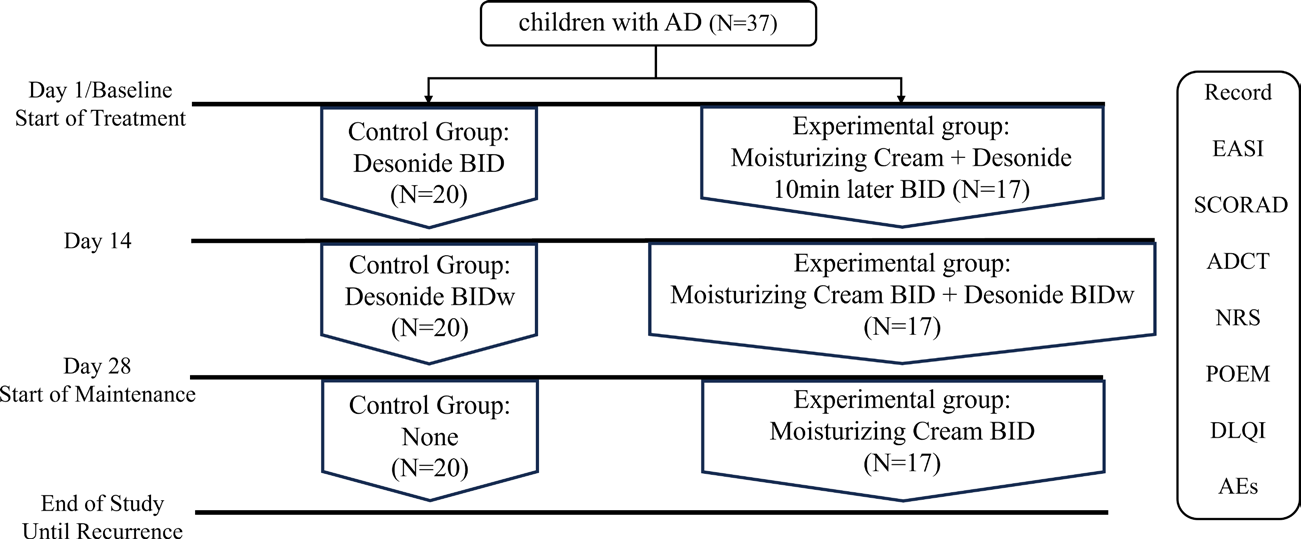
Study design. AD = atopic dermatitis, ADCT = Atopic Dermatitis Control Tool, AEs = Adverse events, BID = twice daily, BIDw = twice daily every weekend, DLQI = Dermatology Life Quality Index, EASI = Eczema Area and Severity Index, NRS = Numeric Rating Scale, POEM = Patient-Oriented Eczema Measure, SCORAD = Scoring Atopic Dermatitis.

This prospective study was approved by the Clinical Research and Experimental Animal Ethics Committee of Zhongshan People’s Hospital (No. K2023-097) and was registered with the Chinese Clinical Trial Registry (No. ChiCTR2300074553). Our study estimated that the overall clinical effectiveness of the soothing moisturizing repair cream combined with desonide is between 95% and 98%. Set the expected target value to be 96%, assuming that the clinical effectiveness of desonide alone in this study is 60%, set alpha = 0.025 (unilateral), and the test effectiveness is 80%. Through the Power Analysis & Sample Size software calculation, it is expected that 34 subjects will be required. Considering 15% shedding, this study intends to include about 40 subjects. A total of 37 children with AD at Zhongshan People’s Hospital from June to November 2023 were enrolled. Informed consent was obtained from all patients/guardians. The participants were randomly divided into an experimental group of 17 and a control group of 20 (Fig. 1). The study was conducted in compliance with the principles of Good Clinical Practice and the Helsinki Declaration. Basic information and the EASI, Numeric Rating Scale, Patient-Oriented Eczema Measure, Dermatology Life Quality Index, and ADCT scores were also recorded.

Inclusion criteria: fulfilling the essential criteria of Williams’ diagnostic standards (main criteria plus 3 or more secondary criteria) and obtaining informed consent and commitment to adhere to follow-up until the end of the study.

Exclusion criteria: allergy to desonide or soothing moisturizing repairing cream; treatment with corticosteroids or antihistamines within 2 weeks prior to the study; severe exudative or eroded skin lesions; concomitant acute infections; and inability to adhere to medication regimen and follow-up as per medical advice.

Withdrawal or dropout criteria: withdrawal of informed consent by the participant; request to exit the trial for any reason by the participant; and loss to follow-up of the participant.

### 2.2. Intervention

All the applied topicals followed the fingertip unit rule. Experimental group: initial application of moisturizing cream (Heling Pharm Guangzhou Co., Ltd, Guangzhou, People’s Republic of China) to the affected area, followed by desonide cream (Huapont Pharm Co., Ltd, Chongqing, People’s Republic of China) 10 minutes later twice daily (for 2 weeks), and then moisturize with cream twice daily and desonide twice daily only on weekends (for 2 weeks), the maintenance treatment period with moisturizer alone until recurrence. Control group: treated solely with desonide cream twice daily (for 2 weeks), then use desonide cream twice daily only on weekends (for 2 weeks), then maintenance treatment period with nothing until recurrence. Follow-up was performed at weeks 4, 8, and 12 (±3 days), respectively, during the maintenance period.

### 2.3. Efficacy indicators

Treatment efficacies were evaluated on days 0, 7, 14, and 28. Patients were instructed to take regular photographs during treatment, observe prognosis and recurrence, and record the occurrence, duration, treatment methods, and outcomes of any adverse reactions. The use of antihistamines and other topical corticosteroids was prohibited during the treatment and maintenance period. Efficacy indicators include: EASI: a composite score based on the severity of skin lesions in different areas and their proportions relative to the total body surface area; itch intensity: assessed using the Numeric Rating Scale for pruritus; Patient-Oriented Eczema Measure score; Dermatology Life Quality Index score; and ADCT score. Adverse drug reactions recorded during the treatment include itching, stinging, erythema, dryness, skin pigmentation, and skin atrophy.

The evaluation was conducted using the SCORAD (Scoring Atopic Dermatitis) scoring method, where a lower score indicates a better therapeutic effect. The therapeutic index is calculated as the SCORAD reduction index = ([initial total score − total score at each follow-up]/initial total score) × 100%. Therapeutic effectiveness is determined based on the percentage decrease in the total score. Cure is defined as a therapeutic index ≥ 90%; significant effectiveness is a therapeutic index of 60% to 89%; improvement is a therapeutic index of 20% to 59%; and ineffectiveness is a therapeutic index < 20%. The cure rate is calculated as (number of cured cases/total number of cases) × 100%, and the rate of significant effectiveness is (number of cured cases + number of significantly effective cases)/total number of cases × 100%.

### 2.4. Statistical methods

Data processing was conducted using Statistical Package for Social Sciences software (version 19.0). Quantitative data, conforming to a normal distribution, are expressed as mean ± standard deviation (x ± s). Comparisons between groups were made using independent sample *t* tests or Wilcoxon tests. Categorical data are expressed as percentages (%), with intergroup comparisons conducted using chi-square χ^2^ tests. The response rate of the AD children in the 2 groups is 95% confidence interval (CI) calculated; if there is a statistical difference between the 2 groups and the lower line of the 95% CI of the rate difference is >0, the statistical superiority is established; that is, the experimental group is better than the control group. The log-rank test was used to compare the maintenance period remission rate and its recurrence time between the 2 groups. A *P* value of <0.05 was considered statistically significant.

## 3. Results

### 3.1. Basic information

In the experimental group, there were 8 males and 9 females, with an average age of 45.1 ± 35.2 months. The control group consisted of 12 males and 8 females, with an average age of 30.4 ± 45.8 months. There were no statistically significant differences between the 2 groups before treatment (Table 1).

**Table 1:** Comparison of general information between the 2 groups of children.

Group	n	Gender, n (%)		Age (mo)	EASI	NRS	POEM	DLQI	ADCT
		Male	Female						
Control	20	8	12	30.4 ± 45.8	14.3 ± 8.9	4.0 ± 2.4	10.6 ± 5.0	1.8 ± 2.1	8.1 ± 3.9
Experimental	17	8	9	45.1 ± 35.2	19.0 ± 11.8	5.2 ± 2.5	11.0 ± 5.3	3.2 ± 2.0	9.8 ± 4.7
χ^2^/t/w		0.208		210.500	240.000	1.511	0.265	273.000	1.262
*P*		.648		.107	.173	.140	.792	.063	.215

ADCT = Atopic Dermatitis Control Tool score, DLQI = Dermatology Life Quality Index score, EASI = Eczema Area and Severity Index, NRS = Numeric Rating Scale, POEM = Patient-Oriented Eczema Measure score.

### 3.2. Efficacy

After 4 weeks of treatment, in the experimental group, the number of patients who achieved cure, significant effectiveness, improvement, and ineffectiveness were 9 (52.9%), 8 (47.1%), 0, and 0, respectively; in the control group, the number of patients who achieved cure, significant effectiveness, improvement, and ineffectiveness were 0, 12 (60%), 7 (35%), and 1 (5%), respectively. None of the patients reached a cure in the control group, and all the patients in the experimental group achieved effectiveness. The cure rate in the experimental group was higher than that in the control group. The overall cure rate was 52.9% with a 95% CI (27.8%–77%) in the experimental group and was 0 with a 95% CI (0%–16.8%) in the control group. The significant effectiveness rate was 100% (95% CI, 80.5%–100%) and 60% (95% CI, 36.1%–80.9%) in the experimental and control groups, respectively; data were shown in Table 2.

**Table 2:** Overall response between 2 groups after 4 weeks of treatment.

Overall response, n (%)	Experimental (N = 17)	Control (N = 20)
Cure	52.9 (9/17)	0
Significant effectiveness	47.1 (8/17)	60.0 (12/20)
Improvement	0	35.0 (7/20)
Ineffectiveness	0	5.0 (1/20)
Rate of significant effectiveness, % (95% CI)	100 (80.5–100.0)	60 (36.1–80.9)
Cure rate, % (95% CI)	52.9 (27.8–77.0)	0 (0.0–16.8)

CI = confidence interval.

**Figure 2.**
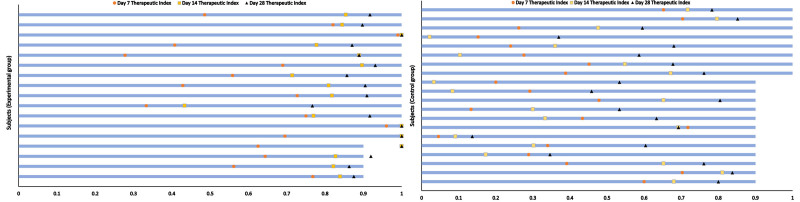
Therapeutic index of each subject in control and experimental group. Short blue lines: 0–2 years group; long blue lines: 2–12 years group.

The therapeutic index of each subject in the control and experimental groups is shown in Figure 2; the circle, square, and triangle indicate the day 7, 14, and 28 therapeutic indexes, respectively. The short blue lines indicate the 0 to 2 years group, and the long blue lines indicate the 2 to 12 years group. The experimental group showed that the majority of patients have achieved a cure at the end of the treatment period, and the 0 to 2-year-old group seems to have a better treatment effect than the 2 to 12-year-old group in both the experimental and control groups.

**Figure 3.**
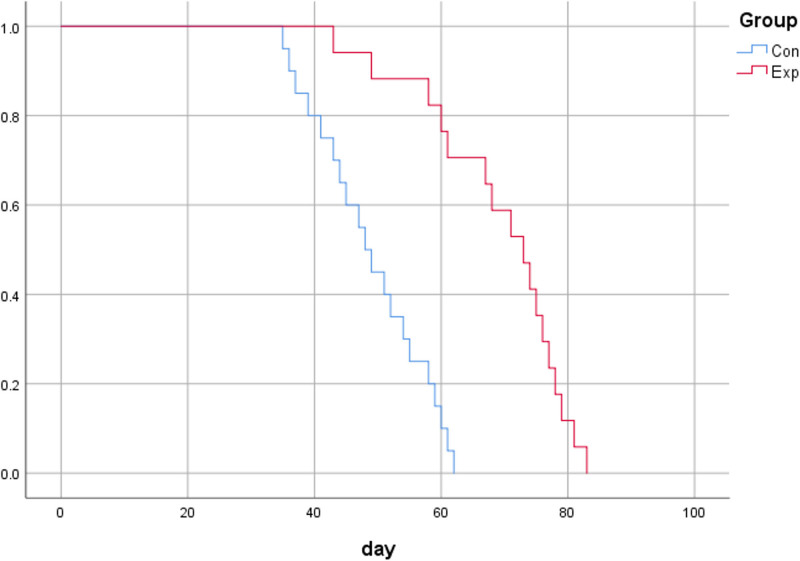
Kaplan-Meier survival estimation of remission rate at each time point in the maintenance period of children with atopic dermatitis.

The Kaplan-Meier survival estimation of remission rate was shown in Figure 3; the estimated average maintenance time was 48.8 days (95% CI, 44.98–52.62) for the control group and 69 days (95% CI, 63.60–74.40) for the experimental group, and the estimated median maintenance time was 48 days (95% CI, 43.62–52.38) for the control group and 73 days (95% CI, 64.93–81.07) for the experimental group, with log-rank χ^2^ = 24.56, *P* = .000; the difference was statistically significant.

The Exp for disease remission in the experimental group compared with the control group was 0.108 (95% CI, 0.04–0.30) in the Cox regression model adjusted for age and sex.

### 3.3. Adverse events

No severe adverse reactions were observed in both the experimental and control groups. In the control group, 2 cases of itching and rash recurrence were reported 1 week after discontinuation of desonide. In the experimental group, 1 patient had an aggravated transient rash during use and then insisted on relieving the symptoms of medication; 1 child had local skin tingling during use; these 2 children had aggravated transient itching, and the 2 medicines were added together. The appearance of a transient adverse reaction is normal and reasonable. There were no statistically significant differences between the 2 groups in terms of adverse reaction incidence and disease recurrence rate.

## 4. Discussion

Our study demonstrates that the use of desonide cream alone as the treatment for pediatric AD in the control group leads to significant improvement in disease severity. However, when compared to the experimental group of children treated with a combination of desonide cream and moisturizing cream, the clinical efficacy is significantly inferior after treatment. Our study shows the clinical effectiveness of the experimental and control groups was 100% and 60%, which achieves the estimated effective rates of 96% and 60%, respectively. This indicates the scientific validity and effectiveness of this study. The estimated average and median maintenance time in the experimental group was nearly twice that of the control group, indicating that the combination medication could significantly delay the recurrence time of children with AD.

AD is commonly presented as a chronic, inflammatory, and recurrent skin condition, often accompanied by symptoms such as skin dryness and itching. Multiple studies have confirmed that the use of topical moisturizers can help repair the skin barrier and effectively reduce disease severity and flare-ups.^[9–11]^ Children, as a special population, may also experience respiratory allergic reactions like asthma, and some may even continue to have periodic relapses into adulthood, severely affecting the physical and mental health of both affected children and their family members. Therefore, when selecting topical medications for pediatric AD, it is crucial to prioritize characteristics such as high efficacy, safety, and low side effects. Desonide cream is a nonhalogenated, low-potency corticosteroid. As a traditional corticosteroid medication, it possesses anti-inflammatory, antiallergic, anti-itch, and exudate-reducing properties. It achieves these effects by inhibiting antibody synthesis within the body, reducing the release of allergic mediators like histamine, and alleviating fever, redness, and swelling caused by local noninfectious inflammation, thus mitigating inflammation and itching reactions.^[12]^ Wollenberg et al^[13]^ further emphasize that children should choose suitable moisturizers based on their individual conditions, and children should use moisturizers of at least 100 g per week.

In recent years, medical skincare products, such as therapeutic cosmetics, have gained recognition for their ability to repair the skin barrier and assist in the treatment of AD. Products such as Yuze Skin Barrier Repair Cream, Vinoona Moisturizing Cream, and Magic Time Baby Multi-Effect Repair Essence Cream, among others,^[14]^ have been confirmed to offer these benefits. Their common effects include protecting the skin barrier and enhancing the body’s moisture and hydration retention capabilities.

The Soothing Moisturizing Repairing Cream selected in this study is primarily composed of 4 major ingredients: 4-tert-butylcyclohexanol, triterpenol and sterols/triterpene esters, sugar isomers, and glycyrrhizal A. 4-Tert-butylcyclohexanol, acting as an antagonist for the Transient Receptor Potential Vanilloid 1 receptor subtype, functions as a pain reliever by inhibiting calcium ion channels, suppressing the action of nicotine, voltage-gated calcium channels, and transient receptor potential cation channel subfamily M (melastatin) member 8. It effectively soothes sensitive skin, inhibits prickling and burning sensations, and provides rapid relief from itching.^[15]^ Triterpenol and sterols/triterpene esters, extracted from avocado fruit oil, possess properties that soothe the skin, strengthen the barrier, inhibit histamine activity, reduce inflammation, and enhance skin barrier function. Sugar isomers, derived from edible corn sugar, bind to lysine in keratin proteins and promote short-term moisturization and moisture retention in the stratum corneum, thereby improving squamation in the medium to long term. Research has shown that products containing sugar isomers can provide deep skin moisturization for up to 72 hours, increasing skin’s water retention capacity by 38%. Glycyrrhizal A, as a phosphodiesterase-4 inhibitor, is an extract from licorice root. It effectively reduces the release of pro-inflammatory cytokines, alleviates erythema, regulates cellular inflammation and lipid metabolism, and inhibits the release of various inflammatory mediators or cytokines such as interleukin-1β and interleukin-6, thereby playing a crucial anti-inflammatory role and improving skin barrier function.

In the “stepped” treatment and management model of AD recognized domestically and internationally, emollients are an important non-drug treatment intervention for all skin lesions and non-skin lesions in children with AD.^[16]^ The use of a sufficient amount of repairing emollients in AD treatment to repair the damaged skin barrier has been recognized and widely implemented by doctors, children, and their parents. However, in clinical practice, there is still a lack of clinical research data on the long-term use effect and safety of emollients in the maintenance period of children with AD. Our study demonstrates that the combination of a soothing moisturizing repairing cream and desonide cream for the treatment of pediatric AD leads to improvements in clinical efficacy in children, as well as a reduced rate of adverse reactions after a 4-week treatment period. During the maintenance period, the use of moisturizers can prolong the time to recurrence. This combination therapy is more effective for children aged 0 to 2 years compared to those aged 2 to 12 years.

However, our study has several limitations. First, the patients included in the study were limited to Chinese children, indicating a need for future research involving diverse ethnicities. Second, the duration of treatment in our study was relatively short (lasting <12 weeks). Third, this study was conducted with a limited number of children at a single center, and subsequent studies will aim to include comprehensive analyses of data across various age groups and multicenter collaborations.

## Acknowledgments

The authors thank all the investigators involved in this study as well as the children and their caregivers who participated in this study.

## Author contributions

**Conceptualization:** Chen Li.

**Methodology:** Chen Li, Yueqing Chen.

**Writing – review & editing:** Chen Li.

**Data curation:** Yuping Zhang, Yueqing Chen.

**Formal analysis:** Yuping Zhang.
